# A novel curcumin oil solution can better alleviate the motor activity defects and neuropathological damage of a Parkinson’s disease mouse model

**DOI:** 10.3389/fnagi.2022.984895

**Published:** 2022-07-29

**Authors:** Xiwen Geng, Hao Zhang, Minghui Hu, Xiaoyu Liu, Min Han, Jinlu Xie, Zifa Li, Feng Zhao, Wei Liu, Sheng Wei

**Affiliations:** ^1^Experimental Center, Shandong University of Traditional Chinese Medicine, Jinan, China; ^2^Key Laboratory of Traditional Chinese Medicine Classical Theory, Ministry of Education, Shandong University of Traditional Chinese Medicine, Jinan, China; ^3^Shandong Provincial Key Laboratory of Traditional Chinese Medicine for Basic Research, Shandong University of Traditional Chinese Medicine, Jinan, China; ^4^Innovative Institute of Chinese Medicine and Pharmacy, Shandong University of Traditional Chinese Medicine, Jinan, China; ^5^Institute of Pharmaceutics, College of Pharmaceutical Sciences, Zhejiang University, Hangzhou, China; ^6^Key Laboratory of Vector Biology and Pathogen Control of Zhejiang, School of Medicine, Huzhou Central Hospital, Huzhou University, Huzhou, China; ^7^Department of Encephalopathy, The Second Affiliated Hospital of Shandong University of Traditional Chinese Medicine, Jinan, China

**Keywords:** curcumin, Parkinson’s disease, protective effect, bioavailability, dopaminergic neuron

## Abstract

Curcumin has been reported to improve or prevent movement disorders in Parkinson’s disease (PD); however, its low bioavailability is the biggest obstacle to its application. To optimize the limited efficacy of curcumin and to improve its protective effects against PD, we prepared and tested a novel curcumin oil solution. *In vivo* imaging was used to confirm that the curcumin oil solution has higher bioavailability than curcumin alone. To test its motor effects on 1-methyl-4-phenyl-1,2,3,6-tetrahydropyridine (MPTP)-induced movement disorders, behavioral tests, including the open-field test, pole test, rotarod test, and automated gait analysis were used. Finally, pathological evaluation using immunohistochemistry and western blotting analysis was done. Encouragingly, the behavioral test findings exhibited a better protective effect against MPTP-induced movement disorders. In addition, it had a greater protective effect on dopaminergic neurons in the compact part of the substantia nigra along with the PD process according to pathological evaluation. This novel curcumin oil solution may provide a new choice for PD prevention as a dietary supplement or clinically assisted treatment based on its better bioavailability and efficiency.

## Introduction

Parkinson’s disease (PD) is a disease of aging that is characterized by selective loss of dopaminergic neurons in the substantia nigra. The global burden of PD on society and individuals has more than doubled over the past two decades (Bloem et al., 2021). Considering the progressive condition of PD and the limitations of existing treatment methods, potential strategies or early intervention drugs that can play a protective role are urgently required. Consequently, the discovery of new interventions that could significantly reverse or slow down the neurodegenerative process of PD is crucial to improving treatment against this disease.

Curcumin is an active natural compound isolated from turmeric (*Curcuma longa*) that has anti-inflammatory, antioxidant, anti-apoptotic, radical scavenging, and antimicrobial effects (Prasad et al., 2021). As a treatment for PD, curcumin represents a promising therapeutic and nutraceutical choice that causes pharmacological effects (El Nebrisi, 2021). Unfortunately, it is also accompanied by low bioavailability, which is the biggest obstacle to its application. The absorption of curcumin is very poor and its metabolism is fast, which leads to low plasma and tissue drug levels that therefore limits drug efficacy (Tabanelli et al., 2021). Here, we report a new curcumin formulation as an oil solution that exhibits better bioavailability and ideal effects against 1-methyl-4-phenyl-1,2,3,6-tetrahydropyridine (MPTP)-induced movement disorders. This may provide a preventive and disease-modifying therapeutic method for PD.

## Materials and methods

### Animals

**FIGURE 1:**
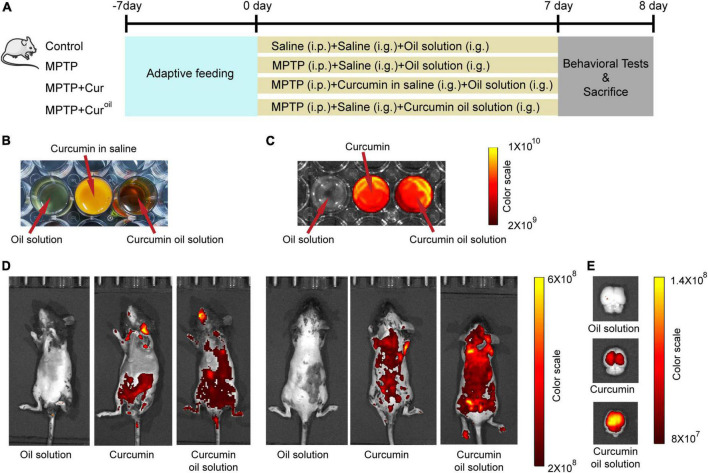
The curcumin oil solution showed higher bioavailability in mice. **(A)** The schedule of the experiment assignment. **(B)** The appearance of the oil solution without curcumin, curcumin in saline and prepared curcumin oil solution. **(C)** The fluorescence images of oil solution without curcumin, curcumin and curcumin oil solution. **(D)** The *in vivo* imaging of mice 1 h after the administration with oil solution without curcumin, curcumin in saline, and curcumin oil solution. **(E)** The fluorescence images of isolated brain tissues from mice 1 h after the administration with oil solution without curcumin, curcumin in saline, and curcumin oil solution. In C-E, the excitation and emission wavelengths were 430 and 509 nm, respectively.

Male C57BL/6J wild type mice (8 weeks old), weighing approximately 15–20 g, were used in this study. All mice purchased from Beijing Vital River Laboratories (Beijing, China) were housed with *ad libitum* access to food and water under an automatically controlled 12-h light/dark cycle at 21 ± 1 ^°^C and 55% relative humidity. All care and experimental procedures were carried out according to the requirements of the National Institutes of Health Guide for the Care and approved by the Animal Experiment Ethics Committee of Shandong University of Traditional Chinese Medicine (No. SDUTCM20210312001). The mice were randomly divided into four groups: control, MPTP, MPTP + Cur, and MPTP + Cur^oil^. The experimental schedule is shown in Figure 1A.

### Curcumin oil solution preparation

Curcumin (purity >98%; C805205) was purchased from Macklin (Shanghai, China). To prepare the oil solution, 0.16 g curcumin, 0.8 g lecithin from soybean (Macklin, L812366), 1.2 g ethoxylated hydrogenated castor oil (Perfemiker, Shanghai, China, PA43980) and 2.5 g glyceryl tridodecanoate (Macklin, G810435) were mixed and stirred adequately. The appearance of the oil solution without curcumin, curcumin in saline, and prepared curcumin oil solution is displayed in Figure 1B.

### Model and drug treatment

To establish a mouse model of PD, mice in the MPTP, MPTP + Cur, and MPTP + Cur^oil^ groups received 30 mg/kg/day MPTP (Sigma, Saint Louis, MO, United States, M0896) intraperitoneal injection for seven consecutive days, and the control animals received an equivalent dose of 0.9% NaCl saline. Simultaneously, mice in MPTP + Cur group were administered 120 mg/kg/day curcumin suspended in 0.9% NaCl saline through intragastric administration once daily before MPTP injection, and mice in the MPTP + Cur^oil^ group received intragastric administration of curcumin oil solution (curcumin dosage = 120 mg/kg/day) (Du et al., 2012; Pan et al., 2012). For comparison, mice in the control, MPTP, and MPTP + Cur groups received intragastric administration of an equivalent volume of oil solution without curcumin. In the whole modeling process, there was no death due to the MPTP injection or the intragastric administration.

### Behavioral testing

On the day after the last drug administration, the mice were tested using the open-field test (OFT), pole test, rotarod test, and automated gait analysis to evaluate the model and drug effect. To prevent interference, each animal was subjected to a single behavioral test (*n* = 13 for each group in each test).

#### Open-field test

Mice were placed into a 50 cm × 50 cm square enclosure and a camera was used to record the motion trail for 6 min with the XR-Super Maze tracking system (Shanghai Xinsoft Information Technology, Shanghai, China), as described in our previous article (Zhang et al., 2020). The total distance, average speed, maximum speed, and distance in the central area (one-ninth of the square) were used to evaluate the motion function.

#### Pole test

This test was conducted with a vertical pole 50 cm long (1 cm diameter) with a small ball (2.5 cm diameter) at the top. On the day before the test, the animals underwent training in three trials. Mice were placed on the top ball and allowed to climb along the pole. The official test was repeated five times, and the time to descend was averaged, with a maximum duration of 120 s. If the mouse could not leave the top ball for descent, the result was considered to be 120 s. If the mouse fell midway, the test was cancelled and retested (Tanimura et al., 2019).

#### Rotarod test

In this test, the training was performed for six consecutive days before the test using a rotarod device (UGO Basile, Italy) with a 3 cm diameter rod. The training was performed at a constant speed (12 rpm) over 60 s, and the test was performed at an accelerated speed (4–80 rpm over 600 s) and repeated four times at 10 min intervals.

#### Automated gait analysis

The CatWalk system (Noldus Information Technology, Wageningen, Netherlands) was used for this analysis. Before the test, a 6-day consecutive training session was performed. Mice were placed in the arena, and the treadmill started at a speed between 5 and 80 cm/s according to the animal’s adaptive capacity until they could run through the treadmill. The speed of the test was 10 cm/s, and data were collected four times from each animal. The average speed, maximum contact area, stands, and swing speed were calculated for analysis using the CatWalk XT 10.5 software (Dalla Vecchia et al., 2018; Chaprov et al., 2021).

### *In vivo* imaging

To observe the biodistribution of curcumin and our oil solution, optical fluorescence imaging was conducted using an *In vivo* Imaging System (PerkinElmer, Waltham, MA, United States). Mice were intragastrically administered curcumin or a curcumin oil solution (curcumin dosage = 120 mg/kg). Control animals received intragastric administration of an oil solution without curcumin. One hour after administration, the mice were anesthetized with isoflurane, and fluorescence images were acquired at excitation and emission of 430 and 509 nm wavelengths, respectively, according to the autofluorescence characteristic of curcumin (Lubtow et al., 2019). Simultaneously, brain tissues were removed from another group of mice 1 h after drug administration and fluorescence images were acquired (Kang et al., 2016). The drugs used in this part were examined using the same fluorescence wavelengths, as shown in Figure 1C. The software built into the spectral unmixing algorithm was used to avoid high autofluorescence.

### Immunohistochemistry

To visually evaluate the dopaminergic neuron degeneration in the compact part of the substantia nigra (SNc) caused by MPTP injection and the protective effect of curcumin or the curcumin oil solution, brains were isolated from animals treated using intracardial perfusion with 0.01 M phosphate-buffered saline (PBS) and 4% paraformaldehyde as reported previously (Zhang et al., 2021). Frozen sections (40 μm) were collected and examined with tyrosine hydroxylase (TH) staining (1:200 primary antibody: Cell Signaling Technology, mAb58844) and an avidin horseradish peroxidase diaminobenzidine kit (DAB; CWBIO, Beijing, China, CW2069S).

### Western blot

To quantitatively analyze TH protein expression in the SNc. First, the brains were rapidly collected from mice and immediately placed in pre-cooling PBS buffer. Then, 1-mm coronal sections containing the SNc were obtained using a McIlwain tissue slicer and a 15 gauge needle was used to excise the bilateral nuclei precisely. Tissues were homogenized in extraction buffer, ultrasonicated, and centrifuged to obtain the supernatant, as described in our previous report (Geng et al., 2021). The protein concentration of the tissues was detected using a BCA quantitative analysis kit (Beyotime, Shanghai, China; P0012S). 20 μg sample proteins were separated by 10% sodium dodecyl sulfate polyacrylamide gel electrophoresis (SDS-PAGE) and transferred to polyvinylidene fluoride (PVDF) membranes (Beyotime; FFP24) that were blocked with 5% non-fat milk for 1 h and incubated with 1:1000 anti-TH primary antibody as mentioned above and 1:5000 secondary antibody (Affinity, Changzhou, China; S0001). We used glyceraldehyde-3-phosphate dehydrogenase (GAPDH) as an internal reference with 1:5000 primary antibody (Proteintech; 60004-1-Ig) and 1:5000 secondary antibody (Affinity; S0002). The enhanced chemiluminescence reagent western blotting system (Solarbio Science & Technology, Beijing, China, PE0010) was used to visualize the protein band with the BioRad imager (BioRad, Hercules, CA, United States).

### Statistics

Data are calculated as the mean ± standard error of the mean. Statistics were conducted using Prism 8.0.2. Comparisons among groups were performed using one-way analysis of variance (ANOVA) with Tukey’s multiple comparisons test after the Kolmogorov–Smirnov test for normality. If the equal square deviation assumption failed, the Brown–Forsythe and Welch ANOVA tests were used. Statistical results were considered significant when *p* < 0.05.

## Results

### The curcumin oil solution showed higher bioavailability in mice

*In vivo* imaging of mice showed that, compared to curcumin, our curcumin oil solution had a higher biodistribution in the animal body (Figure 1D) and isolated brain tissue (Figure 1E) one h after drug administration.

### The curcumin oil solution showed a better protective effect against 1-methyl-4-phenyl-1,2,3,6-tetrahydropyridine-induced movement disorders

**FIGURE 2:**
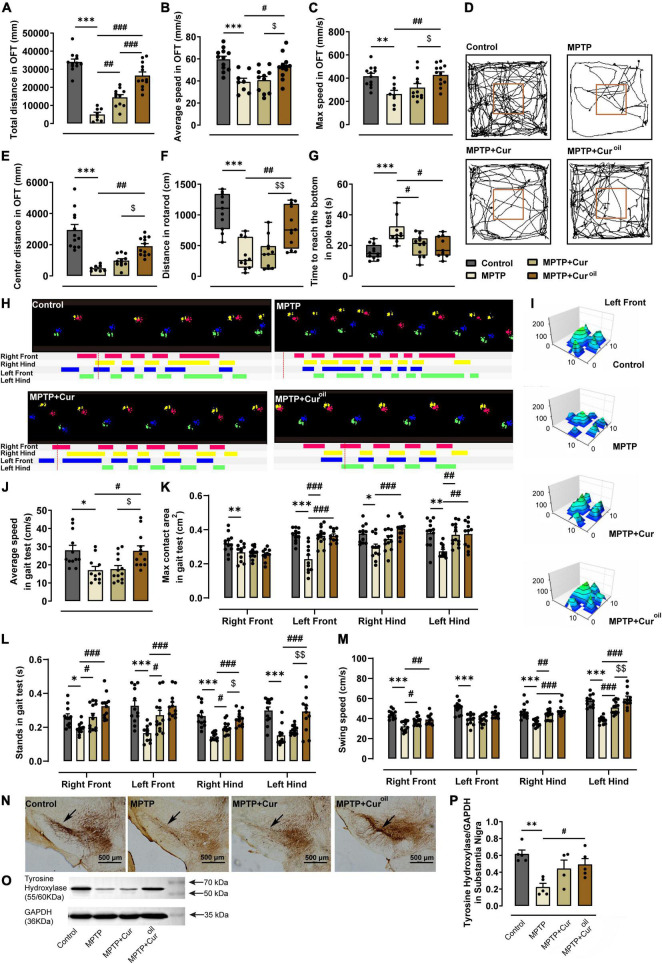
The curcumin oil solution showed a better protective effect against MPTP-induced movement disorders and dopaminergic neuron degeneration in SNc. **(A–C,E)** The statistical results of the open-field test. **(D)** The representative movement paths in the open-field test from mice of each group. **(F)** The statistical results of the rotarod test. **(G)** The statistical results of the pole test. **(H)** The representative footprint track from mice of each group in the gait test. **(I)** The representative 3D view of the left front footprint from mice of each group in the gait test. **(J–M)** The statistical results in the gait test. **(N)** The representative figures of brain slice TH immunohistochemistry from mice of each group. The black arrows show the compact band of substantia nigra. **(O)** The representative protein expression bands from the western blot experiment. **(P)** The statistical result of the relative gray value of the protein bands in the western blot experiment. *p < 0.05 compared to control group, ^**^p < 0.01 compared to control group, ^***^p < 0.001 compared to control group, ^#^*p* < 0.05 compared to MPTP group, ^##^*p* < 0.01 compared to MPTP group, ^###^*p* < 0.001 compared to MPTP group, ^$^*p* < 0.05 compared to MPTP + Cur group, ^$$^*p* < 0.01 compared to MPTP + Cur group.

As shown in Figures 2A–E, mice in the MPTP group showed lower total distance (*p* < 0.001), average speed (*p* < 0.001), maximum speed (*p* = 0.0065), and central distance (*p* < 0.001) than control mice in the OFT. Compared with the MPTP group, curcumin administration only reversed the total distance (*p* = 0.0039), but the novel curcumin oil solution reversed all alterations (*p* < 0.001, *p* = 0.0303, *p* = 0.0032, and *p* = 0.0012, respectively). Similar results were obtained in the rotarod test (Figure 2F). MPTP resulted a significant decline in the distance on the rotarod (*p* < 0.001), and the curcumin oil solution protected this effectively (*p* = 0.0014), but curcumin failed. In the pole test (Figure 2G), MPTP resulted in an increase in time taken to reach the bottom (*p* < 0.001), and mice in the MPTP + Cur group (*p* = 0.0161) and MPTP + Cur^oil^ group (*p* = 0.0139) used less time to reach the bottom compared to the control group.

To evaluate movement functions intensively, automated gait analysis was performed for animals from the four groups. Figure 2H shows a typical foot track of the animals from each group. We can conclude that MPTP resulted in disordered steps and unstable gait, which could be protected by the curcumin oil solution. Statistical results showed that MPTP reduced the average speed of mice (*p* = 0.0156) in the gait test and induced a reduction in the maximum contact area of the four feet (right front *p* = 0.0087, left front *p* < 0.001, right hind *p* = 0.0125, left hind *p* = 0.0020) and a significant decrease in stands (right front *p* = 0.0474, left front *p* < 0.001, right hind *p* < 0.001, left hind *p* < 0.001) and swing speed (right front *p* < 0.001, left front *p* < 0.001, right hind *p* < 0.001, left hind *p* < 0.001). As shown in Figures 2H–M, curcumin administration reversed the maximum contact area of the left front (*p* < 0.001) and left hind feet (*p* = 0.0044), but the curcumin oil solution reversed it for the left front (*p* < 0.001), right hind (*p* < 0.001), and left hind feet (*p* = 0.0044). For the stands in the gait test, curcumin increased the stands of the right front (*p* = 0.0490), left front (*p* = 0.0248), and right hind (*p* = 0.0488) feet, but curcumin oil solution also increased the stands of the left hind feet (*p* < 0.001, *p* < 0.001, *p* < 0.001, *p* < 0.001) and showed more significant differences than curcumin. From Figure 2I, we can intuitively observe the ideal protective effect of curcumin oil solution on the footprint from the 3D view.

### The curcumin oil solution protected the 1-methyl-4-phenyl-1,2,3,6-tetrahydropyridine-induced dopaminergic neuron degeneration in substantia nigra

From TH immunohistochemistry, we could clearly observe SNc dopaminergic neuron degeneration in the brain of the MPTP group, which was characterized by the loss of the compact band of the substantia nigra (Figure 2N). The same conclusion could be obtained from the western blot results shown in Figure 2O where TH protein expression was decreased by MPTP compared to that in the control group (statistical results in Figure 2P showed *p* = 0.0026). The curcumin administration failed to perform its neuron protective effect against the MPTP toxicity on dopaminergic neuron in SNc, but the curcumin oil solution reversed the alteration whether from the histological or the protein expression evidence (*p* = 0.0395).

## Discussion

We prepared a novel curcumin oil solution that showed more efficient bioavailability and a better protective effect against MPTP-induced movement disorders and dopaminergic neuron degeneration in the SNc. Compared with the traditional curcumin drug, this new curcumin oil solution exhibited better protective behavioral and neuropathological effects in a mouse model of PD.

In previous studies, curcumin has been evaluated to improve or prevent movement disorders in PD, but the evidence is not comprehensive enough (Bhat et al., 2019; Rabiei et al., 2019; Abrahams et al., 2021; El Nebrisi, 2021). In this study, through behavioral tests including the OFT, rotarod test, pole test, and automated gait analysis, we provided comprehensive and solid evidence regarding the effect of our novel curcumin oil solution on the prevention of movement disorders caused by MPTP toxicity. These tests are widely used assessment tools to evaluate movement functions, and detailed gait damage assessment results in their high sensitivity and accuracy (Opara et al., 2017). Moreover, our results provide evidence closely related to clinic practices, as gait analysis is regarded as one of the most accurate and detailed markers for diagnosis and PD symptom monitoring (di Biase et al., 2020).

To optimize the limited efficacy of curcumin due to its low bioavailability, several new dosage forms, especially some nanoparticles, have been reported and many of them were also targeted against the PD effect. For example, a recent study fabricated curcumin nanoparticles with human serum albumin as a nanocarrier and demonstrated a preventive effect on the progression of PD (Yavarpour-Bali et al., 2019). However, most of these studies focused on the preparation and characterization of drug dosage forms, and high-quality behavioral evaluation of the drug effect was not sufficient. There is rarely a combination of the pathological drug effects of the novel curcumin forms against PD. In addition, most of the nanoparticles are complicated and expensive to produce, which limits their widespread clinical applications. Here, the novel curcumin oil solution prepared using a convenient and low-cost method improved bioavailability (Figure 1). The oil solution we chose showed the bioavailability improvement characteristics probably because the oil provided a protective layer to prevent the degradation of digestive fluid and peripheral drug metabolism, promoted absorption efficiency, ultimately increased blood-brain barriers transmittance. On the other hand, in the oil solution, curcumin could present a more dispersive status than the poorly solubility in water, which may optimize the drug molecule accumulation (Yan et al., 2021).

More importantly, we also provided solid evidence regarding the effect of the drug on preventing the PD process with comprehensive behavioral and neuropathological tests (Figure 2). Specifically, the curcumin oil solution protected against impairments in movement speed, running distance, foot contact area, stands, and swings. And it protected against the most typical pathological change, the dopaminergic neuron degeneration in the SNc (Bloem et al., 2021). We also performed a clinical experiment using this new curcumin dosage, which is not shown in this report. As mentioned above, this study may provide a new choice for PD prevention as a dietary supplement or disease clinical-assisted treatment based on its better bioavailability and efficiency.

However, this study has a limitation in that the mechanism of action of this curcumin oil solution is not explained. As there have been many reports about curcumin against PD, we assume that it could possibly reverse neuron degeneration in SNc and the dopaminergic system disorders in brain according to our findings, improve neuronal mitochondrial function (Abrahams et al., 2021), prevent α-synuclein aggregation (Chetty et al., 2021) or regulate some immune and inflammatory factors (Mollazadeh et al., 2019), which will be verified in our next study. Moreover, about the drug curcumin, we just evaluated its distribution *in vivo* and the brain using the *In vivo* Imaging System but failed to effectively discover the drug metabolism and processes across the blood-brain barrier.

## Data availability statement

The datasets presented in this study can be found in online repositories. The names of the repository/repositories and accession number(s) can be found in the article/supplementary material.

## Ethics statement

The animal study was reviewed and approved by the Animal Experiment Ethics Committee of Shandong University of Traditional Chinese Medicine (No. SDUTCM20210312001).

## Author contributions

FZ, WL, and SW designed the study. XG, HZ, and MHu performed the experiments, analyzed the data, and wrote the manuscript. XL, MHa, JX, and ZL provided essential assistant through this work. All authors reviewed the final manuscript.
